# Evidence That SOX2 Overexpression Is Oncogenic in the Lung

**DOI:** 10.1371/journal.pone.0011022

**Published:** 2010-06-10

**Authors:** Yun Lu, Christopher Futtner, Jason R. Rock, Xia Xu, Walter Whitworth, Brigid L. M. Hogan, Mark W. Onaitis

**Affiliations:** 1 Department of Surgery, Duke University Medical Center, Durham, North Carolina, United States of America; 2 Department of Cell Biology, Duke University Medical Center, Durham, North Carolina, United States of America; University of Barcelona, Spain

## Abstract

**Background:**

SOX2 (Sry-box 2) is required to maintain a variety of stem cells, is overexpressed in some solid tumors, and is expressed in epithelial cells of the lung.

**Methodology/Principal Findings:**

We show that *SOX2* is overexpressed in human squamous cell lung tumors and some adenocarcinomas. We have generated mouse models in which Sox2 is upregulated in epithelial cells of the lung during development and in the adult. In both cases, overexpression leads to extensive hyperplasia. In the terminal bronchioles, a trachea-like pseudostratified epithelium develops with p63-positive cells underlying columnar cells. Over 12–34 weeks, about half of the mice expressing the highest levels of Sox2 develop carcinoma. These tumors resemble adenocarcinoma but express the squamous marker, Trp63 (p63).

**Conclusions:**

These findings demonstrate that Sox2 overexpression both induces a proximal phenotype in the distal airways/alveoli and leads to cancer.

## Introduction

Sox2 is a high mobility group transcription factor essential for early mammalian development and for the maintenance of both pluripotential embryonic stem cells and stem cells in multiple adult tissues [1], [2], [3], [4], [5]. Overexpression of Sox2 has been demonstrated in many different types of solid tumors [6], [7], [8], [9], [10], [11], [12]. Recently, amplification of SOX2 has been described in human squamous cell lung cancers [13], [14], [15]. However, data are lacking regarding a direct in vivo role for Sox2 in oncogenesis.

Sox2 plays important roles in lung epithelium. In the mouse, Sox2 is expressed in lung epithelial cells, both in the embryonic and adult trachea and airway/bronchiolar epithelium [16], [17], [18], [19]. Immunohistochemistry shows Sox2 protein in tracheal basal cells, the p63-positive, undifferentiated progenitors of the mucociliary epithelium [20], and in some, but not all, columnar cells throughout the trachea and adult lung. Deletion of *Sox2* in the adult tracheal epithelium results in a reduction in the proportion of basal cells and inhibits their ability to self renew, both in culture and *in vivo* after injury by exposure to sulfur dioxide (SO_2_) [17]. Sox2 is also important for maintenance of Clara cells, ciliated cells, and goblet cells in the distal airway as Clara cell-specific deletion late in development leads to a cuboidal epithelium lacking these columnar cell types [19].

Given the importance of Sox2 in the maintenance of stem cell phenotypes, its expression in lung epithelium, and its overexpression in a variety of tumors, we sought to define the potential role of Sox2 in mouse and human lung cancer. We find that SOX2 is overexpressed in human lung squamous cell carcinomas compared with adenocarcinomas. We have used a new conditional allele of the Rosa26 locus to overexpress Sox2 either during lung development, using an *SFTPC-Cre* transgene, or in epithelial cells of the adult lung using a Secretoglobin1a1(*Scgb1a1)-CreER* “knock-in” allele [21]. In both models, we find extensive epithelial hyperplasia, especially in the distal bronchioles and adjacent alveoli, which progresses to carcinoma in about half the mice over a period of weeks. In bronchi and at the bronchioalveolar duct junction (BADJ), overexpression of Sox2 leads to development of a pseudostratified epithelium, with p63-positive cells underlying columnar cells. In the alveoli, hyperplasia develops which proceeds to well-differentiated adenocarcinoma in about half of the mice. Many of the hyperplastic cells stain positively for p63 and/or Foxj1, and some cells become ciliated. Finally, in these regions there is upregulation of Cyclin D1, a known target of Sox2 that likely contributes to the abnormal proliferation of these cells.

## Results

### SOX2 is overexpressed in human squamous cell lung cancer

**Figure 1:**
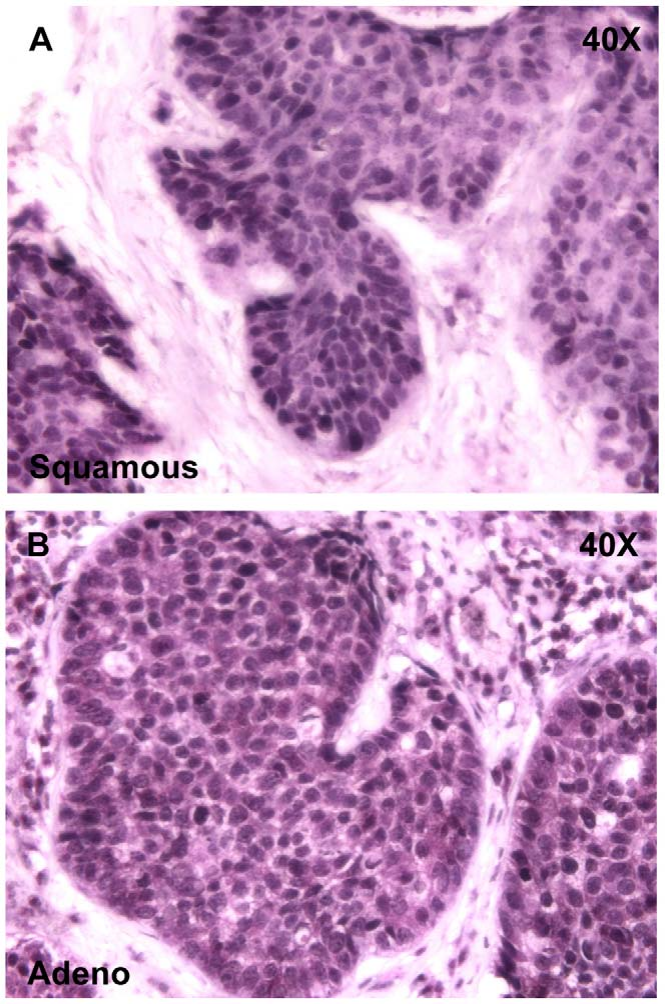
Sox 2 is upregulated in many human squamous cell lung cancers and fewer adenocarcinomas. (A) Sections of a representative squamous cell tumor and (B) adenocarcinoma stained with anti-SOX2. The positively-staining tumor cells are surrounded by negatively-staining stroma.

**Figure 2:**
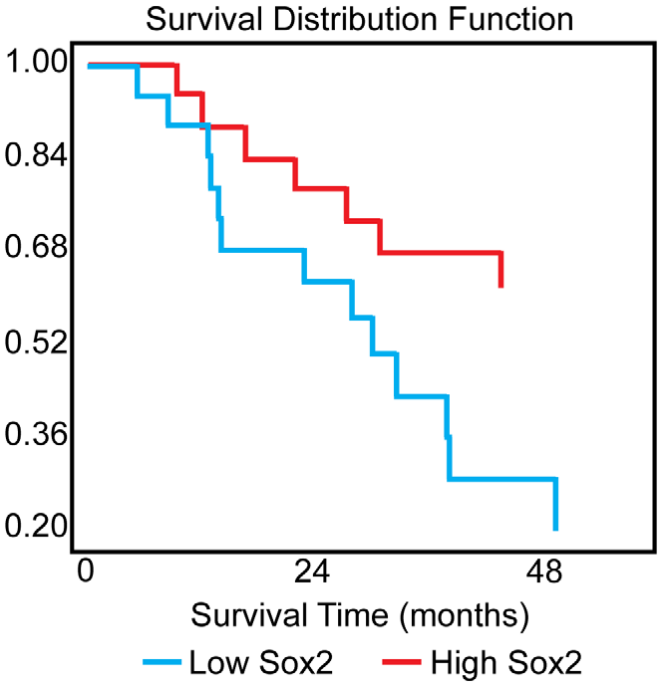
Kaplan-Meier survival curve showing that Sox2 expression in squamous tumors is prognostic of better patient survival times. P = .04 by log-rank test.

We examined the expression of SOX2 in human lung tumors using both transcriptional profiling and immunohistochemistry. An expression microarray dataset of 89 early-stage lung cancers that were resected at Duke University Medical Center was analyzed. Unsupervised clustering of these data using the open-source CLUSTER and TreeView software demonstrates that adenocarcinomas and squamous cell tumors cluster separately (data not shown). Moreover, *SOX2* is highly (5.9-fold) overexpressed in squamous tumors when compared with adenocarcinomas (p<.0001, data not shown). In addition, *SOX2* is expressed in a small subset of adenocarcinomas. These findings were verified using a set of adenocarcinomas and squamous cell cancers from the University of Michigan (n = 281 (130 squamous, 151 adeno)) (data not shown). These findings are consistent with previously-published data from other groups [13], [15]. We also obtained histologic slides from the tumors in the Duke dataset for immunohistochemical analysis. As shown in sections of representative tumors in Fig. 1A and B, 15/20 squamous tumors stain strongly for SOX2. By contrast, 4/20 adenocarcinoma samples stained positively for SOX2 (Table S1). Additionally 17/20 squamous tumors, and 2/20 for adenocarcinomas stained positively for p63 (Trp63), a protein involved in the self-renewal of stratified epithelial progenitors [22] and a frequent marker of squamous cell cancer that clusters closely with *SOX2*. In 14 of 20 squamous tumors, there was positive staining for both Sox2 and p63. No adenocarcinoma stained positively for both SOX2 and p63, although others have demonstrated Sox2+/p63+ staining in up to 12% of human adenocarcinomas [23]. We next asked whether high *SOX2* expression has prognostic significance. Using the squamous tumors from the Duke dataset, tumors with *SOX2* expression above the median had a statistically-significantly better prognosis than those with *SOX2* expression below the median (Fig. 2). However, this could not be repeated using the Michigan squamous cell cancer samples.

### Transgenic overexpression of Sox2 during lung development

**Figure 3:**
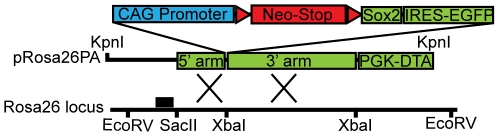
A schematic description of the *Rosa26R-Sox2-IRES-GFP* allele targeting scheme.

**Figure 4:**
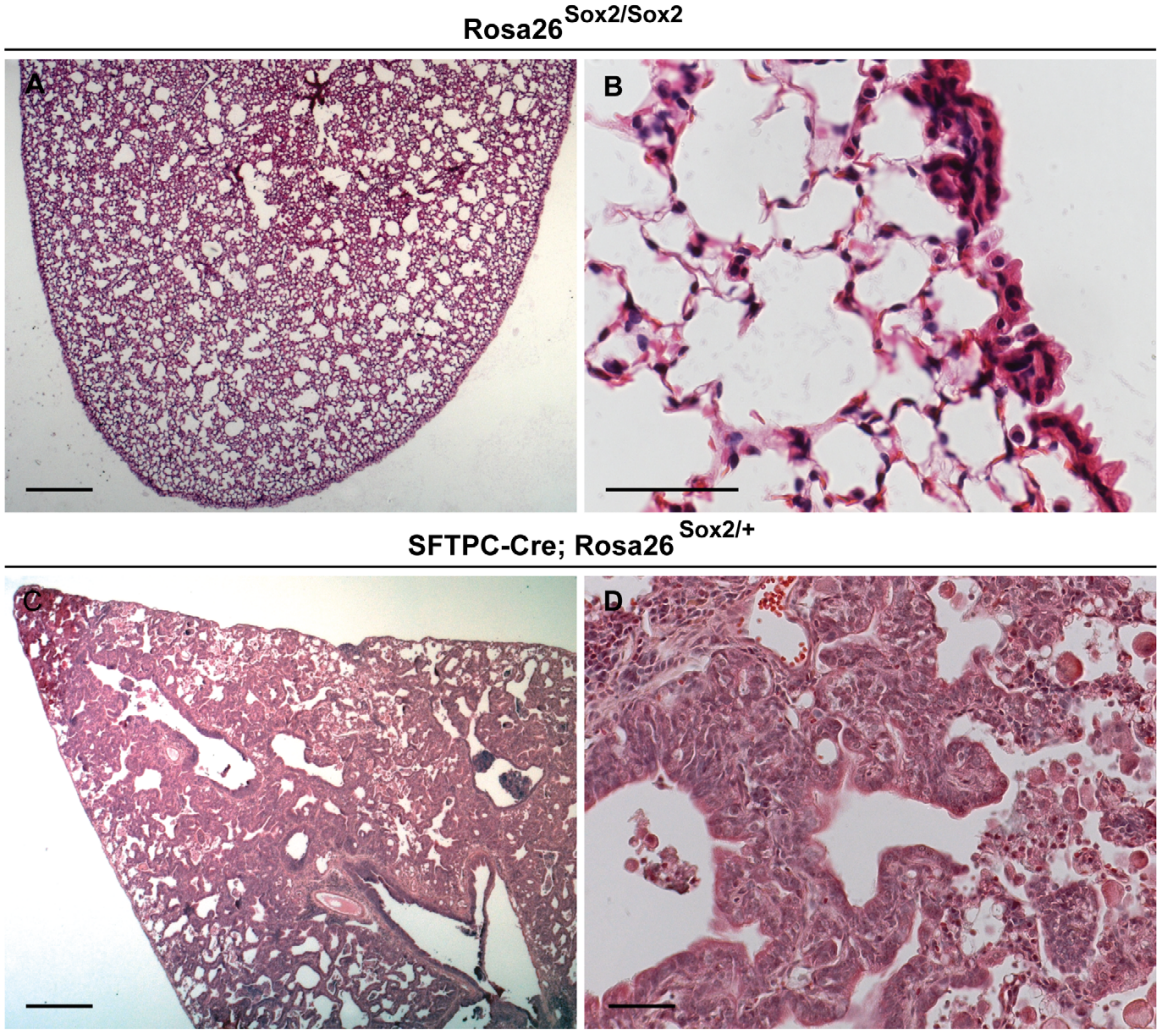
Sox2 overexpression during mouse lung development leads to epithelial hyperplasia and adenocarcinoma. (A) Section of control 15 week post-natal *Rosa26-Sox2-IRES-GFP* lung stained with H&E. (B) High-power region of bronchus and adjacent alveoli of (A). (C) Section of an *SFTPC-Cre; Rosa26-Sox2-IRES-GFP* transgenic mouse lung 15 weeks after birth stained with H&E. (D) A higher power view showing adenocarcinoma. (A,C) Scale bars = 200 microns. (B,D) Scale bars = 50 microns.

To directly test the effect of overexpression of Sox2 *in vivo*, we generated a new conditional allele of the Rosa26 locus (*Rosa26-lox-stop-loxSox2(Gt(Rosa)26Sor^tm1/Sox2/blh^*)(Fig. 3)(hereafter referred to as *Rosa26R-Sox2-IRES-GFP*). In this allele, removal of the stop cassette flanked by loxP sites leads to *Sox2* expression under the control of the Rosa26 locus. To increase the level of *Sox2* transcription after recombination, we inserted a synthetic cytomegalovirus early enhancer/chicken beta actin (CAG) promoter upstream of *Sox2*. For identification of cells in which recombination had occurred, we inserted an internal ribosomal entry-enhanced green fluorescent protein (IRES-GFP) cassette downstream of *Sox2*. To elucidate the effect of Sox2 overexpression in all epithelial cells of the lung during development, we crossed *Rosa26R-Sox2-IRES-GFP* mice with mice carrying a transgene in which Cre is driven by a regulatory region of the human surfactant protein C gene (*SFTPC*). This transgene is expressed from approximately E10.5 in the epithelial cells of the primary buds that will contribute to both the proximal and distal postnatal lung [24]. Most of the *SFTPC-Cre; Rosa26R-Sox2-IRES-GFP* mice were sacrificed within a few weeks of birth with respiratory distress (for numbers see Materials and Methods). Histologic analysis of a mouse that survived to 15 weeks after birth showed very abnormal lung morphology with ectopic expression of p63 throughout the bronchi and alveoli, and regions of epithelial hyperplasia in the bronchi, bronchioles and alveoli as described by others [16]. In addition, as demonstrated in Fig. 4, regions of the lung parenchyma were almost completely replaced by adenocarcinoma. Staining for phospho-histone-H3 reveals increased proliferation of epithelial cells (5–8 per high-power field(hpf)) in the Sox2-overexpressing lungs as compared to controls (very rare cell per hpf) (data not shown).

### Conditional overexpression of Sox2 in the adult lung

We next tested the effect of inducible overexpression of Sox2 in the lungs of adult mice. To do this, we used the *Secretoglobin1a1(Scgb1a1)-CreER* allele in which CreER is present in epithelial cells expressing Scgb1a1 (CC10, CCSP). Previous studies have shown that four injections of tamoxifen every other day, the dose given here, result in recombination in approximately 80% of Clara secretory cells of the airways of the lung extending down to the bronchioalveolar duct junction and in some dual positive Sftpc-Scgb1a1 epithelial cells in the alveoli adjacent to the BADJ [21]. Mice were sacrificed at various times up to 34 weeks after the tamoxifen injections.

We first analyzed the effect of Sox2 overexpression by generating *Scgb1a1-CreER* mice heterozygous for the *Rosa26R-Sox2-IRES-GFP* allele. At 6 weeks after tamoxifen injections, lungs of these mice show no discernible abnormalities. However, by 13 weeks, bronchial epithelial hyperplasia developed near the BADJ. Immunohistochemistry showed that about 50% of the cells in the hyperplastic regions were positive for p63 at this time. In addition, small groups of p63-positive cells were present in the alveoli (data not shown). No p63 positive cells were seen in the airways of control adult mice. At 18 weeks, the hyperplastic regions had not progressed to cancer in these heterozygous mice.

**Figure 5:**
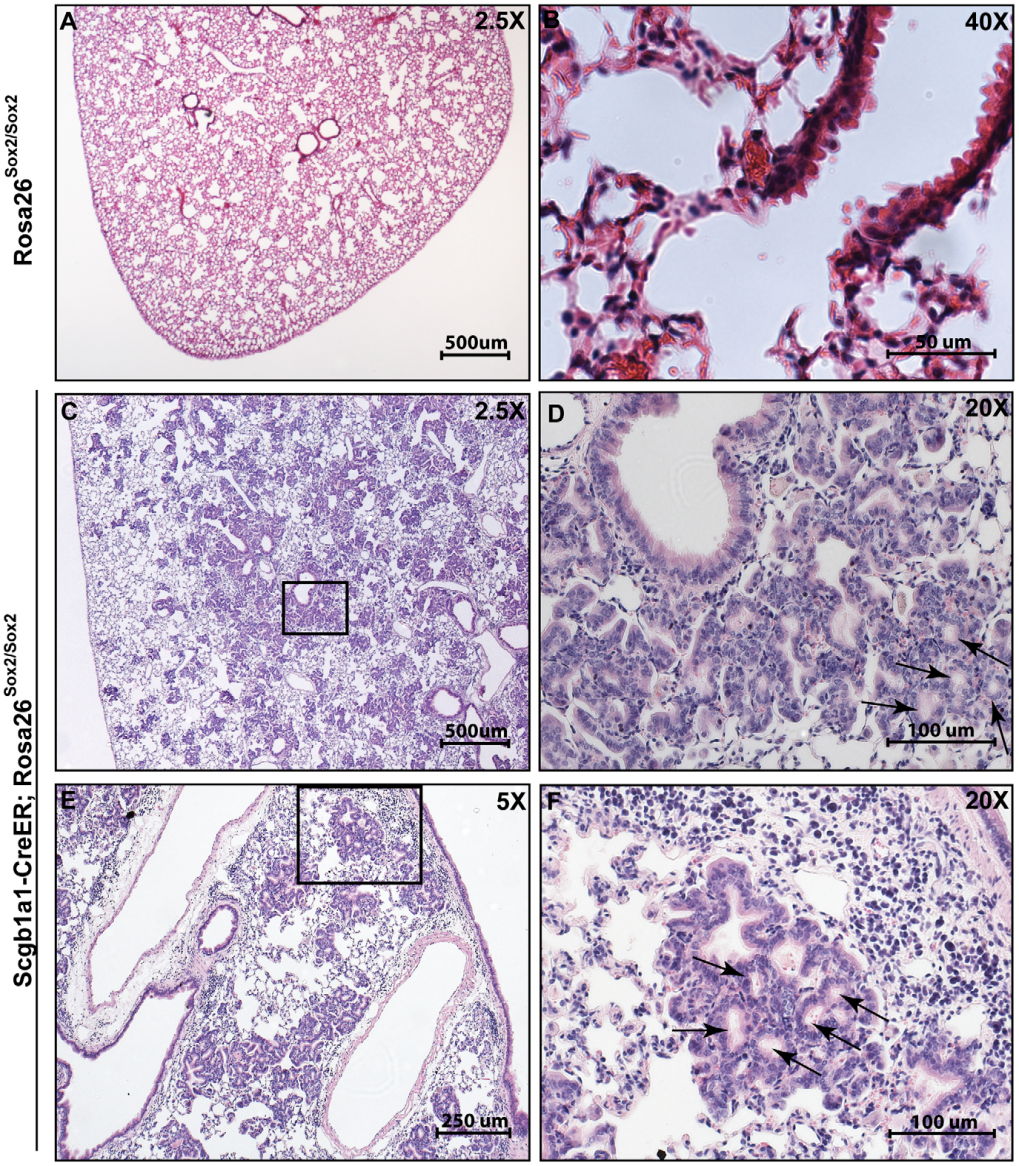
Tumor development in an inducible adult mouse model of Sox2 overexpression in Scgb1a1-expressing (Clara) cells. (A,B) Low-power and high-power magnifications of control homozygous Rosa26-Sox2-IRES-GFP lung stained with H&E. (C–F) Sections through the lung of *Scgb1a1-CreER*; homozygous *Rosa26-Sox2-IRES-GFP* 18 weeks after tamoxifen injection. (C and E) Low-power view showing numerous regions of adenocarcinoma throughout the lung. (D and F) High magnification showing glandular structure formation indicative of adenocarcinoma (arrows). (A,C) Scale bars = 500 microns. (B) Scale bar = 50 microns. (D and F) Scale bar = 100 microns. (E) Scale bar = 250 microns.

**Figure 6:**
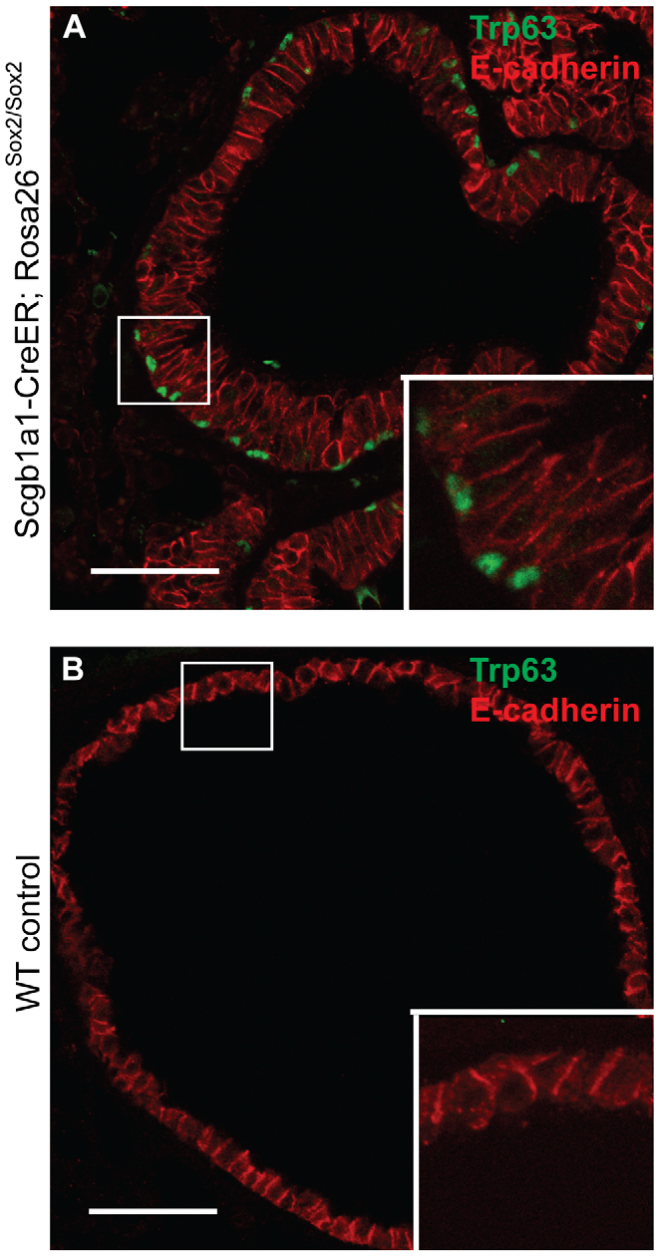
High-power images of bronchi of (A) a *Scgb1a1-CreER; homozygous Rosa26-Sox2-IRES-GFP* and (B) a *homozygous Rosa26-Sox2-IRES-GFP* control mouse lung 18 weeks after tamoxifen injection stained with anti-E cadherin (red) and anti-p63 (green) demonstrating the appearance of a layer of p63-positive cells underlying columnar cells. Scale bars = 50 microns.

In order to assess whether a higher level of Sox2 would lead to cancer, we made the mice homozygous for the *Rosa26R-Sox2-IRES-GFP* allele. These mice were sacrificed at various times from 6 to 34 weeks after the last tamoxifen injection. In all mice examined at early stages (6 weeks after tamoxifen injection), bronchial hyperplasia is seen at and just proximal to the BADJ, and clusters of p63-positive cells develop at the BADJ and in the alveoli (data not shown). Over the next 12–34 weeks, however, both at the BADJ and in the alveoli, this hyperplasia progresses into carcinoma in about half (9/22) of the mice (p<0.0001 when compared to control mice by Chi square). A board-certified human pathologist classified these tumors as well-differentiated adenocarcinoma on standard hematoxylin and eosin staining (Fig. 5 C–F). Relevant histologic features include lepidic growth of the cells, back-to-back glandular-like structure formation, disruption of the normal septal architecture, and the impressively diffuse replacement of normal parenchyma by cancer cells. In addition, the bronchioles of these mice contain regions of pseudostratified epithelium, with p63-positive cells (Fig. 6) underlying columnar cells. However, these cells did not stain positively for the basal cell markers Keratin 14 and Nerve Growth Factor Receptor (NGFR). By contrast, no phenotypic changes are seen in the trachea and large bronchi at any time.

**Figure 7:**
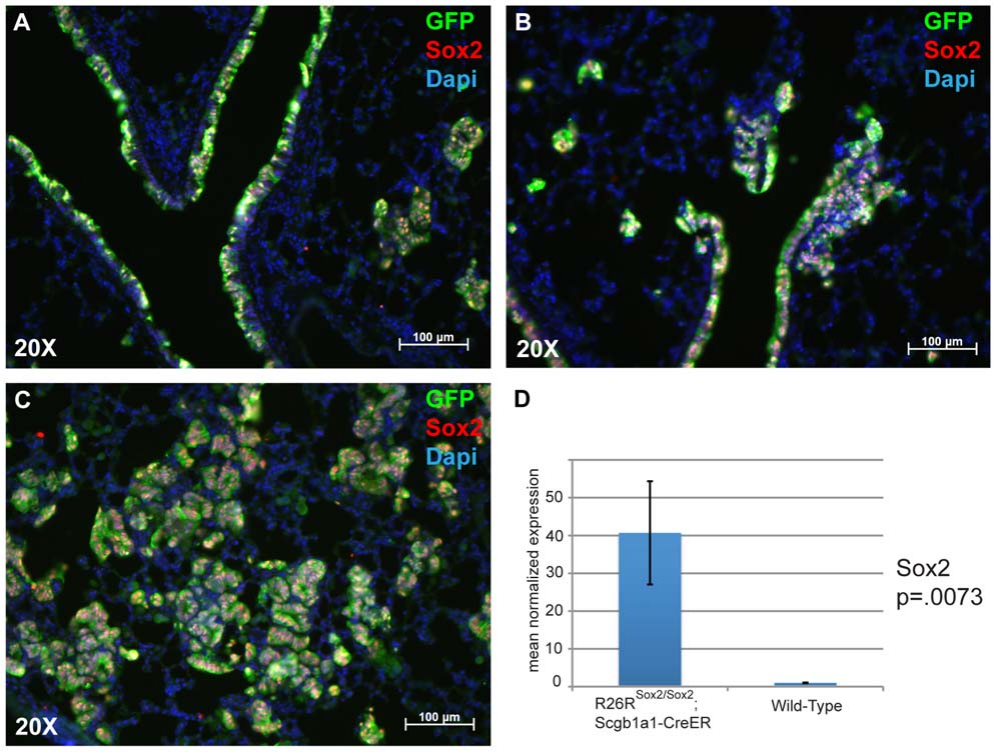
Immunohistochemical and QPCR analysis of Sox2 and GFP expression in tumors and conducting airways. High-power magnifications of *Scgb1a1-CreER; homozygous Rosa26-Sox2-IRES-GFP* lung 18 weeks after tamoxifen injection stained for GFP and Sox2. Concurrent staining of Sox2 (red) with GFP (green) is demonstrated in the conducting airways (A), BADJs (B), and peripheral tumors in the alveoli (C). (D) QPCR shows that Sox2 is overexpressed approximately 40 fold in cells sorted by GFP expression. (A–C) Scale bars = 100 microns.

The level of Sox2 expression in cells after recombination was tested by isolation of GFP-positive cells by FACS from *Scgb1a1-CreER*; *Rosa26R-Sox2-IRES-GFP* lungs. As a control we isolated cells from Scgb1a1-CreER mice homozygous for a *Rosa26R^FGFP^* reporter allele. Quantitative PCR of the two populations showed an increase in mean normalized expression of 40 fold in *Scgb1a1-CreER; Rosa26-Sox2-IRES-GFP* cells vs. wild type (Fig. 7D). Significantly, this level of expression was seen at both 1 week and 34 weeks after tamoxifen administration, and there was no significant difference in expression levels in cells from mice with hyperplasia only compared with those with tumors. These observations suggest that progression of hyperplasia to carcinoma is not due to an increase in the expression level of Sox2 but is more likely associated with secondary changes in the epithelial cells. The fact that only about half of the mice (9/22) develop tumors suggests that modifier genes may be important in tumor progression.

**Figure 8:**
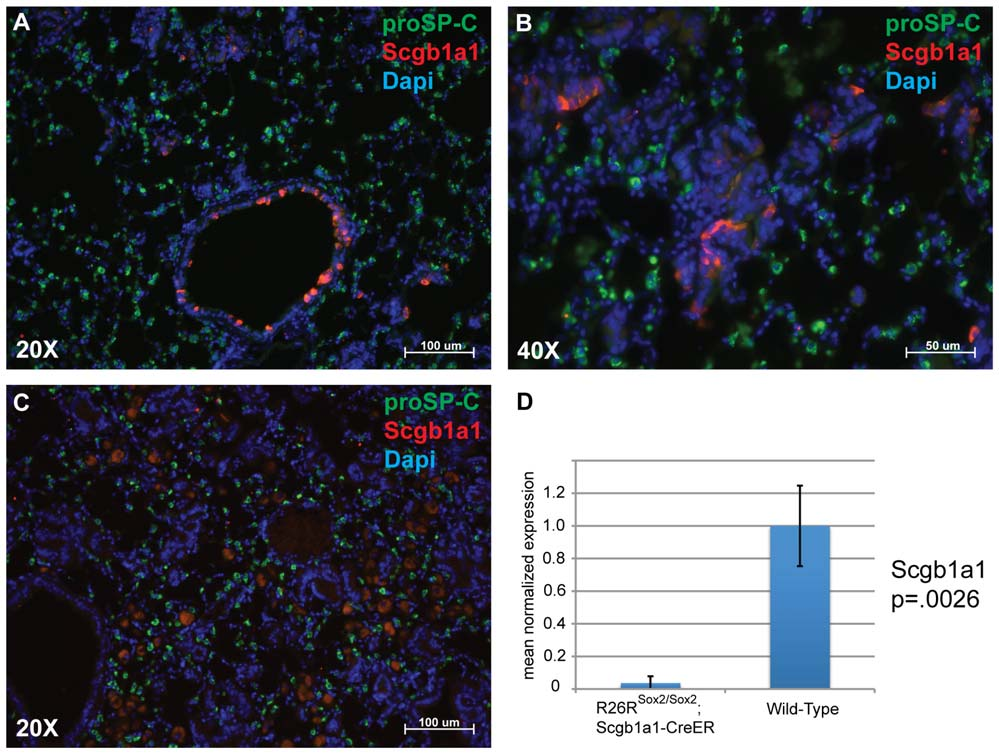
Immunohistochemical analysis of Scgb1a1 and Sftpc (proSP-C) expression in tumors and conducting airways. Scgb1a1 (red) expression is downregulated within the conducting airways by 12 weeks after tamoxifen injection (A). However, expression is initially maintained within the tumors at 12 weeks (B). By 18 weeks, expression of Scgb1a1 is completely lost within the tumors and lung (C). Orange cells within panel C are autofluorescent macrophages. Sftpc (proSP-C) (green) expression is not noted within the tumors at any stage. (D) QPCR confirms the downregulation of Scgb1a1 within *Scgb1a1-CreER; homozygous Rosa26-Sox2-IRES-GFP* lung 18 weeks after tamoxifen injection. (A and C) Scale bar = 100 microns. (B) Scale bar = 50 microns.

**Figure 9:**
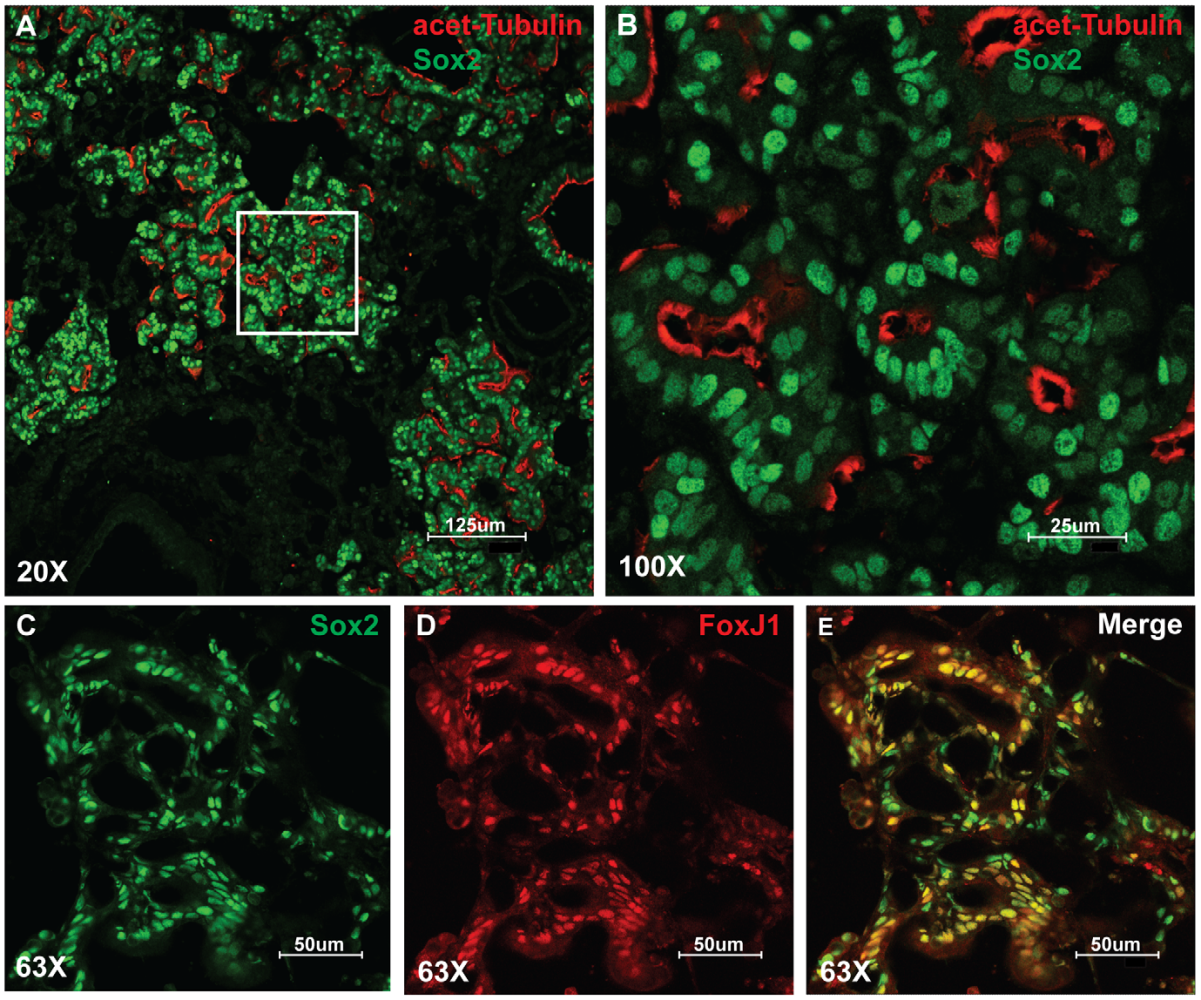
Expression of ciliated cell markers in Sox2 overexpressing tumors. (A and B) Cilia are expressed on the apical surfaces of Sox2 positive (green) tumor cells as shown by acetylated tubulin staining (red). (C–E) FoxJ1(red) is expressed in a subset of Sox2 (green) positive tumor cells. (A) Scale bar = 125 microns. (B) Scale bar = 25 microns. (C–E) Scale bar = 50 microns.

**Figure 10:**
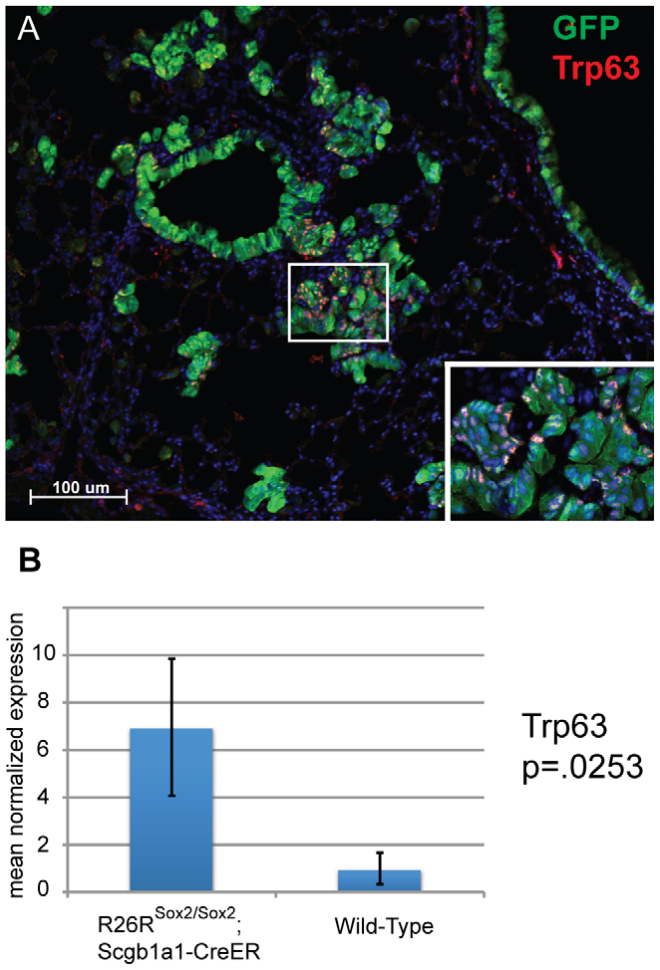
Expression of p63 in Sox2 overexpressing tumors. (A) p63 (red) is expressed in a subset of Sox2 overexpressing tumor cells as marked by GFP (green) expression. (B) p63 upregulation is confirmed by QPCR. (A) Scale bar = 100 microns.

Immunohistochemical analysis of the tumors for bronchial epithelial markers was then performed. As expected, immunohistochemistry of lungs in homozygous *Rosa26R-Sox2-IRES-GFP* mice demonstrates high levels of recombination as measured by GFP expression and Sox2 protein within the conducting airways, BADJs and the alveolar tumors (Fig. 7A–C). Although Sox2 is inducibly overexpressed in Scgb1a1-expressing cells, Scgb1a1 expression gradually decreases over time, eventually becoming completely absent at late stages of tumor development (Fig. 8A–C). This was confirmed by quantitative PCR of sorted cells, which demonstrates that Scgb1a1 expression is diminished 100-fold (Fig. 8D). The tumors do not stain for the type II cell marker Sftpc. Surprisingly, many of the cells that comprise the hyperplastic regions and eventual adenocarcinomas in the alveoli stain positively for the ciliated cell markers Foxj1 and acetylated tubulin (Fig. 9). In addition, approximately 50% of the tumor cells stain positively for the basal cell marker, p63. Again, p63 expression was confirmed by quantitative PCR that showed upregulation in *Scgb1a1-CreER; Rosa26-Sox2-IRES-GFP* cells (Fig. 10).

**Figure 11:**
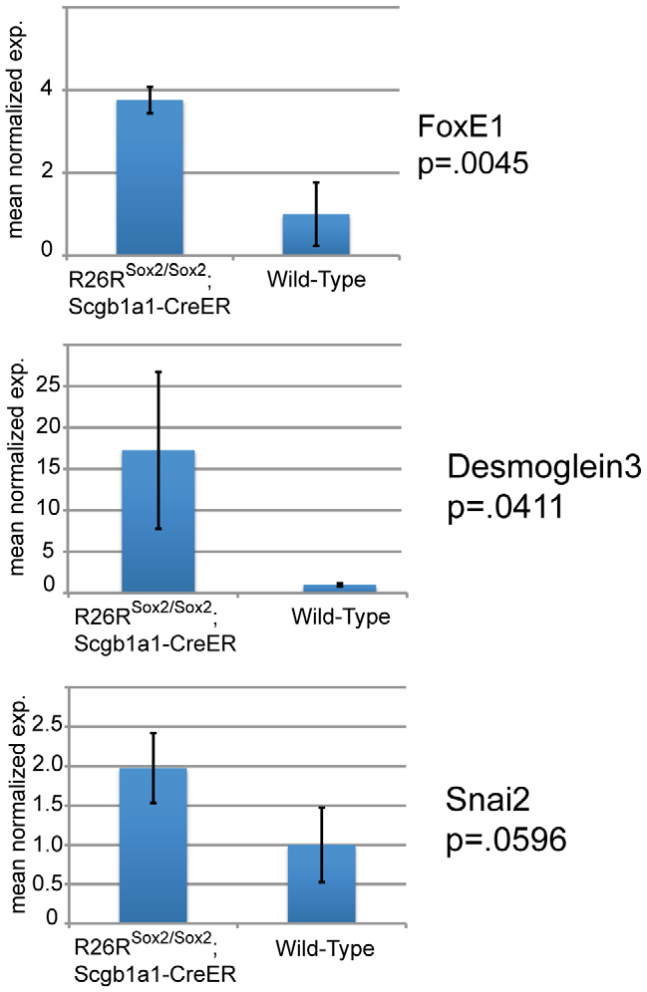
QPCR shows that Sox2 overexpressing cells upregulate a number of squamous cell cancer markers including FoxE1, Desmoglein3, and Snai2.

In order to assess the transcriptional similarity of the alveolar carcinomas to human squamous cell lung cancer, we performed quantitative PCR for additional transcripts that cluster closely with Sox2 in the Duke lung cancer microarray dataset. As Fig. 11 demonstrates, FoxE1 and Desmoglein, are statistically-significantly upregulated in the Scgb1a1-Rosa26-Sox2-IRES-GFP tumor cells compared with wild-type Scgb1a1-positive cells. Snai2 is also upregulated in the tumor cells. Thus, although the tumors were classified as adenocarcinomas, they contain a significant proportion of cells that express squamous cell markers in addition to p63.

**Figure 12:**
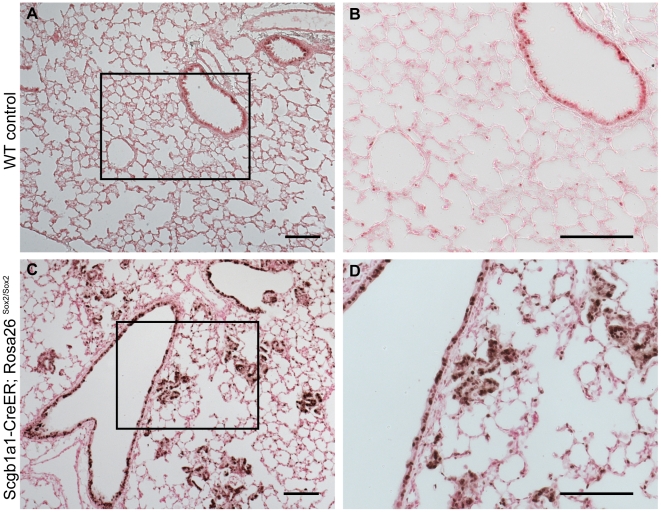
CyclinD1 is highly-expressed in Sox2-overexpressing lungs, and Sox2 overexpression leads to a pseudostratified epithelium. (A) Low-power image of a control lung demonstrating expression of Cyclin D1 restricted to a few bronchial cells. (B) High-power image of (A). (C) Low-power images of anti-Cyclin D1 immunohistochemistry of sections of a *Scgb1a1CreER- Rosa26Sox2-IRES-GFP* transgenic lung 12 weeks after tamoxifen injection demonstrating high expression of Cyclin D1 in hyperplastic alveolar clusters. (D) High-power image of (C). (A–D) Scale bars = 100 microns.

To begin to elucidate the mechanism of Sox2-induced neoplasia, we stained sections of tumors from *Scgb1a1-CreER; homozygous Rosa26R-Sox2-IRES-GFP* lungs for Cyclin D1, a target of Sox2 in breast cancer cell lines [25]. As Fig. 12A–D demonstrates, approximately 90% of hyperplastic cells in the alveoli stain positively for Cyclin D1. Control lungs show Cyclin D1 staining only in some bronchial epithelial cells. This likely explains the increased proliferation of the Sox2-overexpressing cells as Cyclin D1 is known to promote G0/G1-to-S transition in the cell cycle.

**Figure 13:**
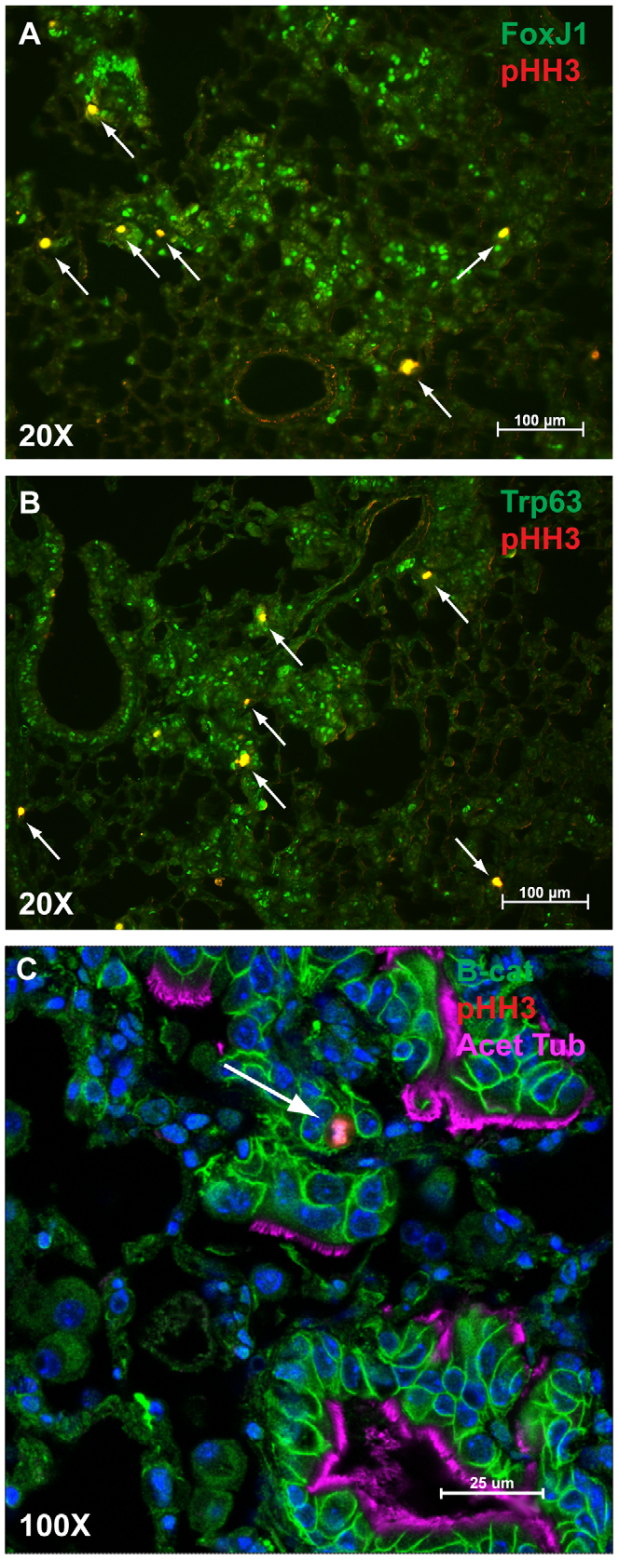
Actively proliferating cells 18 weeks after tamoxifen as marked by Phosphohistone H3 (red) (arrows) staining are both FoxJ1 (green) positive (A) and p63 (green) positive (B). (C) High-power image demonstrating that Phosphohistone-H3-positive cells (red) (arrow) do not stain for acetylated tubulin (purple). Cell membrane is marked by beta-Catenin (green). (A,B) Scale bars = 100 microns. (C) Scale bar = 25 microns.

In order to determine which cells within the tumor were proliferating, we co-stained sections for phosphohistone-H3 and p63 or FoxJ1. Significantly, we found that all phosphohistone-H3 positive cells within the tumors were also positive for p63 and for FoxJ1 (Fig. 13 A–B) indirectly suggesting that the proliferative cells may be progenitors that co-express both transcription factors. As Fig. 13C demonstrates, none of the ciliated cells in the tumors stained positively for phosphohistone-H3.

## Discussion

We have directly demonstrated *in vivo* that overexpression of Sox2 is oncogenic. Current mouse models of lung cancer are almost exclusively adenocarcinoma [26]. In contrast to human tumors, mouse tumors have less stromal response and metastasize less frequently. In our model, Sox2 overexpression in Scgb1a1-positive cells drives development of histologically well differentiated adenocarcinoma with significant squamous cell features including widespread expression of p63, FoxE1 and Desmoglein3. This mixed phenotype is not unique to tumors induced by Sox2. Inducible deletion of the tumor suppressor *Lkb1* along with inducible expression of oncogenic K-Ras leads to adenocarcinoma with squamous features [27] as well. One possibility for this mixed phenotype in our model is that while Sox2 alone can drive expression of some squamous tumor markers, an additional oncogenic stimulus, - “a second hit” - may be required to drive complete squamous differentiation. Noticeably missing from our model are cytokeratins 5 and 14, markers of squamous cell cancer, which may account for the lack of characteristic squamous features on H&E.

We speculate that cell-of-origin may also contribute to differences between tumors arising in these locations and that expression of the conditional *Sox2* allele in other respiratory epithelial cells will lead to tumors of different phenotypes. We are therefore continuing to investigate whether different cell types of origin may result in tumors with more squamous features.

It has been suggested that lung adenocarcinoma may arise from Scgb1a1, Sftpc dual positive putative bronchial alveolar stem cells (BASCs), that reside near the BADJ. Our findings are consistent with the idea that this population is more susceptible to tumorigenesis than the majority of cells within the epithelium in which Sox2 is overexpressed. This is supported by the observation that only hyperplasia develops within the conducting airways while adenocarcinoma forms at the BADJ. However, a model in which BASCs are the only cancer-initiating cell is overly simplistic. We note extensive tumor formation in the alveoli, and evidence from lineage tracing for the presence of cells that contribute descendants to both bronchioles and alveoli *in vivo* is still lacking [21]. We note that human squamous cell tumors have been classified into proximal and distal types [28]. It is likely that the very peripheral tumors develop from Scgb1a1-positive cells that already reside within the alveoli in a different microenvironment than the cells within the bronchioles.

The mechanism of Sox2's oncogenic ability is unclear and will be the focus of future investigation. CyclinD1 expression is clearly induced in our model. The *CCND1* gene has a SOX2-binding sequence in its promoter, and chromatin immunoprecipitation has revealed SOX2 and beta-catenin cooperative binding regulating this promoter in breast cancer cell lines [25]. Directly overexpressing Cyclin D1 in mammary tissue in transgenic mice using the mouse mammary tumor virus (MMTV) promoter leads to breast cancer, demonstrating that Cyclin D1 is sufficient for tumorigenesis [29]. Finally, Cyclin D1 is necessary to effect transformation of cells downstream of specific oncogenic stimuli [30]. Further characterization of the transcriptional targets of Sox2 as well as downstream events in Sox2-transformed Clara cells is continuing.

The prognostic relevance of SOX2 in human tumors is unclear. SOX2 may induce a stem-like transcriptional program and has been demonstrated to be upregulated in poorly-differentiated adenocarcinomas by genomic analyses [31], [32]. A recent study has demonstrated that a SOX2+/p63- immunohistochemical profile correlates with high-grade histology across non-small cell lung cancer subtypes [23]. In the Duke dataset, patients with squamous tumors with expression of SOX2 mRNA above the median enjoyed a better prognosis than those with lower levels of expression. The reasons for this discrepancy are unclear. However, they may relate to differences between tumor types included in the datasets: SOX2 may play different roles in squamous cell tumors and adenocarcinomas. Such issues will be studied further in the future as Sox2 is inducibly expressed in other bronchial epithelial cell types in the mouse.

An important feature of our model is that overexpression of Sox2 leads not only to distal hyperplasia and cancer but also to development of a pseudostratified epithelium with p63-positive cells adjacent to the basal lamina and FoxJ1 cilliated cells reaching to the apical surface. This epithelial metaplasia is seen both proximal to the BADJ and in larger bronchioles/bronchi. This result suggests that high levels of Sox2 can lead to transdifferentiation of Scgb1a1-positive cells (and/or their daughters) into basal-like and ciliated cells. Whether all or only a subset of Scgb1a1-positive cells throughout the intralobar airway are able to undergo this transformation is not yet known. In addition, it will be important to determine whether the p63 isoform expressed is deltaNp63 or TA-p63 and whether p63 and FoxJ1 are direct downstream targets of Sox2. We note that the p63 promoter contains 3 conserved Sox2 consensus sites. Moreover, a recent report demonstrated squamous metaplasia of the alveoli of the lung by overexpression of deltaNp63 downstream of a tetracycline-inducible SftpC promoter [33].

Initially, the expression of cilia within the Sox2 overexpressing tumors seemed to confound Sox2 overexpression in Scgb1a1 positive cells as a model of human cancer. However a recent report suggests that this may not be the case [34]. They report three cases of well-differentiated adenocarcinoma that feature a bland cytological appearance, prominent ciliated cells and express p63. It is very likely that lack of reporting of ciliated adenocarcinomas is not due to their absence in human cancer but due to the simple fact that cilia is not a feature commonly looked for during histology of resected human tumors.

Given the possibility that a proportion of human non-small cell lung cancers arise from SOX2 upregulation, this and future mouse models will be useful in testing existing and new therapeutics for this group of patients. From a translational perspective, characterization of oncogenic stimuli in specific potential cells-of-origin of lung cancer is an important step toward designing individualized therapies for human lung cancer patients.

## Materials and Methods

### Ethics Statement

All mouse work was performed according to the requirements of the Duke University Institutional Care and Use Committee (IACUC). Mouse husbandry, breeding, genotyping, tamoxifen injection, and euthanasia specifically for these experiments were approved via protocol number A226-07-08. Euthanasia was performed by CO2 asphixiation.

### Microarray Analysis

The publicly-available microarray data (accession number GSE3141 NCI GEO) from lung cancer patients resected at Duke was obtained and RMA-normalized using the publicly-available RMAExpress program (rmaexpress.bmbolstad.com/). The expression of SOX2 was compared between adenocarcinomas and squamous cell carcinomas using a two-tailed t test. For Kaplan-Meier analysis, the median expression of SOX2 was determined across squamous cell tumors. The squamous cases were then grouped in a binary fashion with respect to expression of SOX2 above or below the median. SAS Version 9 with Enterprise Guide 4.0 was used to perform the survival analysis with log rank test to determine significant difference.

### Mice

The *SFTPC-Cre* and *Scgb1a1-CreER* lines have been described previously (Okubo et al. 2005; Rawlins et al. 2009b). The *Rosa26R-Sox2-IRES-GFP* and *Rosa26R-FGFP* alleles where generated by inserting a CAG–loxP-STOP-LoxP-Sox2 –IRESGFP-polyA and CAG-loxP-STOP-loxP-eGFP-f-polyA (eGFP-f = farnesylated eGFP) cassettes respectively into a Rosa-acceptor targeting plasmid. Mouse lines where generated by homologous recombination in 129/SvEv embryonic stem cells followed by injection into C57/Bl6 blastocysts. Chimeras were bred for germline transmission. The mice were maintained on a mixed 129/SvEvxC57/Bl6 mixed background. Tamoxifen injections were carried out at 6 weeks of age at a dose of 0.25 mg/kg body weight in corn oil delivered intraperitoneally every other day for four injections. GFP reporter activity (GFP and eGFP-f) is detected only after Cre-mediated recombination of the LoxP sites.

For the *SFTPC-Cre; Rosa26R^Sox2-IRES-GFP/+^* experiments, three mice were moribund at postnatal day (P) 11 and one at P16. Of the remaining mice, one was sacrificed at 12 weeks, and one at 15 weeks.

For the *Scgb1a1-CreER*; *Rosa26R^Sox2-IRES-GFP/+^* experiments, mice were sacrificed at various times from 6 weeks, 12/13 weeks, and 18 weeks after tamoxifen injection. For the *Scgb1a1-CreER*; *Rosa26R^Sox2-IRES-GFP/Rosa26RSox2-IRES-GFP^* experiments, mice were sacrificed and sectioned at various timepoints from 6–34 weeks after tamoxifen injection (Table S1).

Representative sections of the abnormal lungs were reviewed with a board certified lung pathologist with experience in mouse lung pathology.

### Immunohistochemistry

Paraffin sections were stained with the following antibodies: rabbit anti-Sox2 (1∶1500, Seven Hills Bioreagents), mouse anti-p63 (1∶250, Santa Cruz), rat anti-phophohistone-H3 (1∶500, Upstate), goat anti-Scgb1a1 (1∶10,000, kind gift of Dr. Barry Stripp), rabbit anti-Sftpc (pro-SPC) (1∶500, Millipore), chicken anti-GFP (1∶500 Aves Labs), rat anti-E-Cadherin (1∶1000, Zymed), mouse anti-acetylated tubulin (1∶100, Sigma-Aldrich), mouse anti-Foxj1 (1∶1000, eBioscience), rabbit abti-beta Catenin (1∶1000, Sigma-Aldrich), and rabbit anti-CyclinD1 (1∶1000, Cell Signaling). For fresh tumor staining and CyclinD1 staining, biotinylated secondary antibody was used followed detection with the ABC kit (Vectorlabs). For fluorescent staining, Alexa-Fluor coupled secondary antibodies (Invitrogen) were used at 1∶500. Z-stacks of optical sections were captured on a Leica Sp2 laser scanning confocal microscope. Multiple optical sections were scored manually to distinguish cell boundaries.

For human tumor SOX2 analysis, each stained slide was evaluated by 2 observers blinded to histology and microarray expression level. Positive staining was called when greater than 5% of nuclei stained positively.

### Cell Sorting and qPCR

Quantitative PCR (qPCR) was performed on mRNA isolated from Sox2 lung epithelial cells isolated from *Scgb1a1-CreER*; *Rosa26R^Sox2-IRES-GFP/Sox2-IRES-GFP^* and *Scgb1a1-CreER*; *Rosa26R^FGFP/FGFP^* mice. Single cell suspensions of lung epithelial cells were prepared as described elsewhere (Teisanu et.al. 2009). Cells were sorted based on GFP expression using a BD FACSVantage cell sorter. RNA was then extracted from the sorted cells using the Qiagen RNeasy kit, treated with DNase I, and cDNA was synthesized using the Bio-Rad iScript cDNA synthesis kit. qPCR was performed on an Eppendorf Mastercycler ep realplex real-time PCR system using the following Taqman primer probe sets from Applied Biosystems; Sox2 - Mm00488369_s1, Scgb1a1-Mm00442046_m1, Trp63-Mm00495788_m1, Krt14-Mm00516876_m1, and Gusb-Mm00446953_m1. Beta glucuronidase (Gusb) served as the reference control.

## Supporting Information

Table S1 Annotation of microarray and histologic samples.
(0.03 MB XLS)
